# Feasibility of ABLE 1.0—a program aiming at enhancing the ability to perform activities of daily living in persons with chronic conditions

**DOI:** 10.1186/s40814-021-00790-7

**Published:** 2021-02-18

**Authors:** Kristina Tomra Nielsen, Susanne Guidetti, Cecilie von Bülow, Louise Klokker, Eva Ejlersen Wæhrens

**Affiliations:** 1 Department of Occupational Therapy, University College of Northern Denmark (UCN), Selma Lagerløfs vej 2, DK-9220 Aalborg Ø, Denmark; 2 The ADL Unit, the Parker Institute, Copenhagen University Hospital Bispebjerg - Frederiksberg, Nordre Fasanvej 57, Road 8, entrance 19, DK-2000 Frederiksberg, Denmark; 3 The Research Initiative for Activity Studies and Occupational Therapy, Research Unit for General Practice, Department of Public Health, University of Southern Denmark (SDU), J.B. Winsløwsvej 9, DK-5000 Odense, Denmark; 4 Department of Neurobiology, Care Sciences and Society, Division of Occupational Therapy, Karolinska Institutet, Alfred Nobels Allé 23, SE-141 83 Huddinge, Sweden; 5 The Musculoskeletal Statistics Unit, the Parker Institute, Copenhagen University Hospital Bispebjerg - Frederiksberg, Nordre Fasanvej 57, Road 8, entrance 19, DK-2000 Frederiksberg, Denmark

**Keywords:** Everyday life, Rehabilitation, Health care quality, Disability

## Abstract

**Background:**

The “A Better everyday LifE” (ABLE) intervention was developed to accommodate the need of a program addressing ability to perform activities of daily living (ADL) in persons with chronic conditions living at home. During intervention development, it is necessary to evaluate relevant aspects of the feasibility of a program. Thus, the aim was to evaluate the feasibility of content and delivery of ABLE version 1.0.

**Methods:**

A one group pre- and post-test design was applied. Thirty persons with chronic conditions, two occupational therapists (OTs), and five occupational therapy students (OTSs) participated. ABLE 1.0 is an 8-week program consisting of ADL evaluation (session 1); goal setting and reasons for ADL problems (session 2); intervention (sessions 3–7); and re-evaluation (final session), conducted in the clients’ home-setting and local area. Sessions 1–4 and the final session was mandatory.

To evaluate the feasibility of content and delivery, the OTs, after each session, reported on applied intervention component(s), time-use, needed equipment, adjustments, meaningfulness, confidence, progress toward goal attainment, and side effects using registration forms. The clients reported on progress toward goal attainment, meaningfulness, and satisfaction. Clinically relevant improvements in ADL ability were identified using the ADL-Interview (ADL-I) and the Assessment of Motor and Process Skills (AMPS). Goal attainment was evaluated using the Goal Attainment Scaling (GAS).

**Results:**

Twenty clients (67%) completed ABLE 1.0 and received four sessions (median = 4, range 4–7) each lasting between 30 and 94 min. Most frequently applied component was “Changing habits related to task performance”. Generally, OTs reported having the needed equipment. Deviations from the manual were made by omission of GAS and AMPS and less than mandatory number of sessions per client. The OTs reported confidence in delivering the program and the clients perceived the program as meaningful and satisfying, and experienced progress toward goal attainment. Goal attainment was found in 52% of the goals. Sixteen (80%) clients obtained clinically relevant improvements in self-reported or observed ADL ability.

**Conclusions:**

The content and delivery of ABLE 1.0 was feasible. However, the study revealed a need to adjust the recruitment procedure and make minor changes in the intervention manual. A pilot randomized controlled trial (RCT) study is recommended.

**Trial registration:**

The study was registered at ClinicalTrials.gov with registration no. NCT03335709 on November 8, 2017.

**Supplementary Information:**

The online version contains supplementary material available at 10.1186/s40814-021-00790-7.

## Key messages regarding feasibility

1. What uncertainties existed regarding the feasibility?

As the ABLE 1.0 is a newly developed program, several uncertainties exist. The present study aimed at evaluating the feasibility of content and delivery of this first version of the program from the perspectives of clients with chronic conditions and OTs.

2. What are the key feasibility findings?

Overall, the ABLE 1.0 was feasible in terms of intervention content and delivery according to both clients and OTs.

3. What are the implications of the feasibility findings for the design of the main study?

The present feasibility study implied a need for adjusting the recruitment procedure and the intervention manual. A pilot RCT study is recommended to improve recruitment procedures, determine acceptability of randomization, and further monitor adherence before a full scale RCT study is conducted.

## Background

When living with a chronic condition, the ability to perform activities of daily living (ADL) can be affected. This is reflected in a definition stating that chronic conditions “last a year or more and require ongoing medical attention and/or limit activities of daily living” (ADL) [1]. In line with this definition, studies [2–8] indicate that persons living with chronic conditions (including rheumatologic, neurologic, or medical diseases) experience problems related to performance of both personal ADL (PADL) (e.g., bathing, dressing, and eating) and instrumental ADL (IADL) (e.g., cleaning, shopping, and cooking) tasks [9].

There is evidence to support a structured and individualized problem-solving process to address ADL task performance problems [10, 11]. However, evidence for specific interventions is sparse [10, 11] and the interventions evaluated are not always described in detail [12]. Therefore, there is a need to develop and describe an intervention program specifically addressing ADL task performance problems among persons with chronic conditions. Consequently, as part of the research program “A Better everyday LifE,” we developed the first version of the ABLE program (1.0). The program was developed in accordance with the British Medical Research Council’s (MRC) guidance on how to develop and evaluate complex interventions [13]. The guidance comprises four stages; development, feasibility/piloting, evaluation, and implementation. The development of ABLE 1.0 consisted of a literature search, a study aiming at identifying, organizing, and prioritizing ideas on how to improve ADL ability [14], a study exploring decreased self-reported quality of ADL task performance among persons with chronic conditions [15] and two workshops with researchers experienced in intervention development. This led to the conclusion that clients perceive similar problems related to ADL task performance, predominantly increased time-use and physical effort, across diagnoses. This supports employment of a generic approach, i.e., using the same methods (e.g., energy conservation [16]) when addressing particular types of ADL task performance problems (e.g., increased physical effort during cooking) across individuals with similar performance problems but different diagnoses (e.g., COPD or heart failure). At the workshops, the obtained information was discussed, synthesized, and translated into specific intervention components. The development process is described in detail elsewhere [17, 18].

In the second stage of the MRC guidance, a feasibility study is recommended before implementing a full scale randomized controlled trial (RCT) [13, 19]. O’Cathain et al. [20] suggest evaluating selected aspects of feasibility such as (a) content and delivery of an intervention; (b) design, conduct, and processes of an outcome trial; (c) identification and selection of outcomes; and (d) how to measure. Consequently, the focus of the present study was feasibility in terms of content and delivery of the ABLE 1.0 intervention [17].

## Methods

### Aim

The aim of the study was to evaluate the feasibility in terms of content and delivery of an occupational therapy intervention addressing the ability to perform activities of daily living (ADL) in persons with chronic conditions [17]. More specifically to evaluate *intervention development*; *intervention components*; *mechanisms of action*; *perceived value*, *benefits*, *harms*, *and unintended consequences*; *feasibility and acceptability in practice*; and *fidelity*, *reach*, *and dose* (Additional file 1), using the framework by O’Cathain et al. [20]*.*

### Design

A one group pre- and post-test design was applied. Various types of data was collected; data from registration forms, data from ADL evaluations, and data collected based on qualitative interviews [19]. The results of the qualitative interviews will be reported in a separate paper. A study protocol, following the Standard Protocol Items: Recommendations for Intervention Trials (SPIRIT) 2013 Statement [21, 22], was published [17].

### Setting

The study was conducted in a Danish municipality providing rehabilitation services including group-based, diagnosis-specific (e.g., osteoporosis, chronic obstructive pulmonary disease) rehabilitation programs using psychoeducation and physical exercises. The ABLE 1.0 intervention was delivered in the homes and local areas (e.g., local grocery store) of the clients.

### Participants

Persons with chronic somatic conditions (e.g., rheumatologic, neurologic, or medical diseases), hereinafter named clients, fulfilling the following criteria were included: (a) age ≥ 18 years, (b) diagnosed with > 1 chronic condition(s), (c) completed > 1 group-based, diagnosis-specific rehabilitation programs at the municipality > 1 year prior to recruitment, (d) living at home, (e) experiencing PADL and/or IADL tasks performance problems, and (f) motivated for participation. Persons with known substance abuse, acute illnesses affecting ADL task performance, and/or language barriers hindering participation were not eligible. Clients were recruited from June till September 2017. Based on a list of persons who had completed diagnosis-specific rehabilitation programs > 1 year prior to recruitment, a health care worker employed at the municipality called potential participants by telephone. The health care worker was introduced to both the program and the recruitment procedure. A checklist was developed to support the healthcare work during the recruitment process. The checklist included a description of the aim, content and process of the program, and relevant questions to ask to ensure that inclusion criteria were fulfilled. The clients participated in ABLE 1.0 between September 1^st^ and December 19^th^, 2017.

Occupational therapists (OTs) (*n* = 2) with > 2 years of experience working with persons with chronic conditions, calibrated Assessment of Motor and Process Skills (AMPS) raters and employed at the municipality delivered ABLE 1.0. Pre-graduate occupational therapy students (OTSs) (*n* = 5), trained and calibrated as AMPS raters, conducted post-intervention ADL interviews (ADL-I) and AMPS evaluations.

### The ABLE 1.0 program

The ABLE 1.0 is an occupational therapy program informed by two models: the Person-Environment-Occupation (PEO) model [23] presenting occupational performance as doing shaped by the interaction between a person, an environment, and a meaningful and purposeful task (i.e., occupation) and; the Occupational Therapy Intervention Process Model (OTIPM) [24] describing a problem-solving process. Previous studies support applying OTIPM as a structure for intervention programs [25–28] and PEO to organize intervention strategies [29, 30]. The program is generic; using the same methods, when addressing similar types of ADL task performance problems across individuals with different diagnoses.

**Fig. 1:**
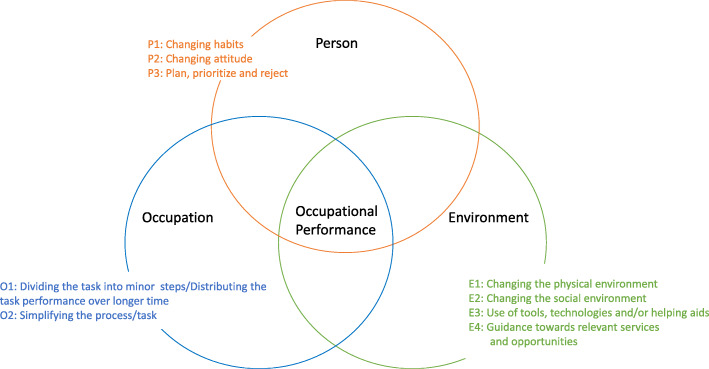
The nine optional intervention components

ABLE 1.0 is an 8-week program aiming at enhancing ADL ability. It is individually tailored based on baseline evaluations and implemented at sites, where the clients typically perform ADL tasks (e.g., home or local area) with the tools and materials usually used [24]. The program consists of session 1: first meeting and occupational therapy evaluation; evaluation of ADL ability based on interview (ADL-I) [31] and observation (AMPS) [32, 33]; session 2: goal setting (GAS) [34, 35] and clarification of reasons for ADL task performance problems; sessions 3–7: interventions aiming at enhancing ADL ability, based on the compensatory model of OTIPM [24], e.g., teaching new ways of doing, using adaptive equipment/assistive technology, modifying physical/social environments. During the intervention sessions, the OT employ one or more of nine optional intervention components organized in a “tool box” according to the PEO model [23] (Fig. 1). Final session: re-evaluation including evaluation of goals and re-evaluation of ADL ability. Sessions 1–4 and the final are mandatory. Thus, while the number of intervention sessions may vary based on the client’s needs, two intervention sessions are considered the minimum. Based on the OT’s reasoning, sessions are carried out face-to-face or by telephone, with or without “homework” between sessions (e.g. trying out new ways of doing).

### Data sources/measurement

#### Demographic and general health data

Using a questionnaire, demographic data characterizing the clients were collected at baseline: age, gender, diagnosis, job situation, civil status, type of help at home, and self-reported general health. General health was assessed using the first question (SF1) of the 36-item Short Form Survey (SF36) [36]. A previous study supports applying the SF1 among persons with chronic conditions [36]. Demographic data on the OTs (age, gender, years since graduation, years working with persons with chronic conditions) and the OTSs (age and gender) were collected at the first training session.

#### Evaluation of feasibility

As described in the protocol paper [17], specific objectives and related data collection methods (Additional file 1) were based on the framework by O’Cathain et al. [20].The feasibility evaluation of content and delivery of ABLE 1.0 was conducted using a combination of data from registration forms and ADL evaluations. Most aspects in the registration forms were evaluated using Visual Analog Scales (VAS) from one to five; 1 = very low degree, 2 = low degree, 3 = fair degree, 4 = high degree, and 5 = very high degree.

##### Registration forms for occupational therapists and occupational therapy students

A registration form (for example see Additional file 2) was developed to be filled in by OT/OTS reporting on deviations from the ABLE 1.0 manual (*intervention development*); components applied, time use, and needed equipment (*intervention components*); if the session (i.e., the applied components) contributed to progress toward goal attainment (*mechanisms of action*); unintended side effects and perceived degree of meaningfulness (*perceived value*, *benefits*, *harms*, *or unintended consequences*); retention, challenges, satisfaction, confidence, and facilitators and barriers (*feasibility and acceptability in practice*); and number of sessions, time use, and dose sufficiency (*fidelity*, *reach*, *and dose of intervention*) (Additional file 1) [17, 20]. Based on the registration forms, the number of clients treated by each OT was also determined.

##### Registrations form for clients

Similarly, a registration form (see example in Additional file 3) was developed to report on aspects related to the feasibility of the intervention from the client’s perspective: if the session contributed to progress toward goal attainment (*mechanisms of action*); the perceived degree of meaningfulness (*perceived value*, *benefits*, *harms*, *or unintended consequences*); to which extent the client perceived to be informed and involved, the perceived degree of satisfaction (*feasibility and acceptability of intervention in practice*) and dose sufficiency (*fidelity, reach and dose of intervention*) (Additional file 1) [17, 20].

##### Data on ADL ability (ADL evaluations)

To explore *mechanisms of actions*, data on ADL ability were gathered to determine the proportion of clients obtaining clinically relevant improvements in ADL ability and the extent to which intervention components contributed to goal attainment. Data on ADL ability comprised self-reported and observed ADL ability measured at baseline and post intervention using ADL-I [31], AMPS [32, 33], and GAS [34, 35].

The ADL-I [31] is a standardized evaluation interview used by OTs to describe and measure self-reported quality of ADL task performance in 47 ADL tasks in terms of physical effort and/or fatigue, efficiency, safety, and independence. During the interview, the person rates *the perceived quality of performance*. The baseline quality of performance ratings form the basis for identification of ADL task performance problems to be prioritized at goal setting. To measure change in self-reported quality of ADL task performance, the 47 quality of performance ratings are transformed into one overall linear (interval scale) ADL-I measure of self-reported quality of ADL task performance, adjusted for the difficulty of the ADL tasks, based on Rasch measurement methods [31]. The measures are expressed in logits (log-odds probability units) [2, 31]. Previous studies indicate that the ADL-I can be used to generate valid and reliable measures of self-reported quality of ADL task performance among persons with various chronic conditions [3, 4, 31].

The AMPS [32, 33] is a standardized observation-based evaluation to measure *observed quality of ADL task performance* in terms of physical effort and/or fatigue, efficiency, safety, and independence. The person chooses and performs at least two relevant standardized ADL tasks of appropriate challenge. Two domains are evaluated; motor skills (16 items) and process skills (20 items). After the observation, the quality of each skill is evaluated on a four-point ordinal scale according to scoring criteria in the AMPS manual [33]. AMPS software [37], based on Many-Faceted Rasch statistics, is used to convert ordinal raw scores into overall linear ADL motor and ADL process ability measures adjusted for task challenge, skill item difficulty, and rater severity. Measures are expressed in logits (log-odds probability units) [32]. ADL motor ability measures below the 2.0 logits competence cutoff indicate increased physical effort, fatigue, and clumsiness during task performance and ADL process ability measures below the 1.0 logit competence cutoff indicate inefficient and potentially unsafe ADL task performance suggesting need for assistance in everyday life [32, 33]. Several studies support reliability and validity of AMPS ADL ability measures among persons with chronic conditions [2, 3, 38–40].

The GAS (34, 35) is a tool for defining and monitoring individual goals. The person is involved in defining goals and describing levels from − 2 to + 2 of goal attainment: “less than expected” (level − 2), “unchanged/actual level” (level − 1), “expected” (level 0), “more than expected” (level + 1), and “much more than expected” (level + 2). Measurable and observable indicators (e.g., independence, duration, frequency) are used, when goals are described. GAS has been found applicable among older adults with multiple chronic conditions living at home [41].

### Procedures

#### Training in relation to data collection and intervention delivery

Initially, OTs delivering ABLE 1.0 participated in a two-and-a-half-day workshop containing lectures on OTIPM, role-play and video demonstrations; workshop on administering ADL-I and GAS; and introduction to the ABLE 1.0 manual and data collection. In addition, the OTs were AMPS re-calibrated prior to initiating data collection. The OTSs participated in the ADL-I workshop and underwent two training sessions on data collection.

#### Data collection

Following each session, clients and OTs/OTSs independently filled out registration forms and clients handed in their forms to the OTs/OTSs in sealed envelopes. To minimize influence from the OTs delivering the intervention, OTSs performed ADL-I and AMPS re-evaluations at the final session. Still, to have the OTs involved in some formal re-evaluation, GAS goals were re-evaluated during the last intervention session.

### Analyses

#### Demographic and general health data

Descriptive statistics were performed using Microsoft Excel software [42]. Ordinal data and data with lack of normal distribution were presented based on median and range, nominal data based on percentages.

#### Feasibility data: registration forms

The number of clients recruited, and the retention rate were presented in a flowchart. The frequency of the implemented components and the median number of minutes used at each session were presented in a histogram. For each type of session, VAS ratings on aspects related to, e.g., confidence, involvement, meaningfulness, progress toward goal attainment, and satisfaction, were presented using medians and ranges.

Answers to questions concerning (a) deviations from the intervention manual, (b) conditions facilitating and/or hindering the delivery of the sessions, (c) potential positive and/or negative side effects, (d) sufficiency of the intervention dose as registered by the OTs, (e) potential positive and/or negative side effects, and (f) sufficiency of the intervention dose as perceived by the clients were summarized and supported by quotes. When relevant, the number of comments on an issue was presented.

#### Feasibility data: data on ADL ability (ADL evaluations)

In accordance with the AMPS manual, proportions of clients with no change (< 0.3 logits), a clinically relevant increase (≥ 0.3 logits) or decrease (≥ − 0.3 logits) in AMPS ADL ability measures were identified [32]. Proportions of clients with no change (change < 0.5 SD) or a clinically relevant change (≥ 0.5 SD) on the ADL-I ability measures were identified [43] based on baseline sample SD. Baseline data (demographic, general health, and observation-based ADL ability; AMPS) of responders (i.e., clients achieving a clinically relevant increase in ADL ability) and non-responders, respectively, were explored in descriptive analyses. Finally, proportions of goals being rated in each of the five goal attainment levels were also identified. All proportions were presented in histograms.

### Study size

For feasibility studies, sample size calculation is not required [44, 45]. Sample size was estimated to 30 participants and justifications here for presented elsewhere [17].

## Results

### Demographic and general health data

**Fig. 2:**
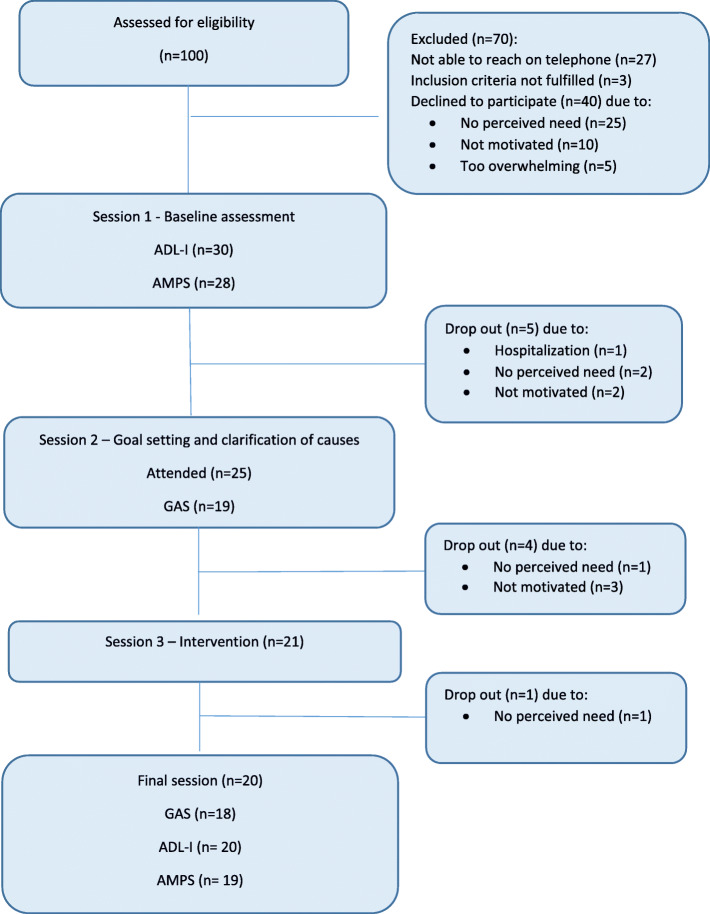
Flowchart

Demographic data of clients, OTs, and OTSs is presented in Table 1. In total, 100 persons with chronic conditions were assessed for eligibility, and 30 (30%) persons were enrolled (Fig. 2). Demographic data indicated variations across diagnoses, gender, and age (Table 1). Baseline mean AMPS ADL motor ability measure was below competence cut-off indicating increased physical effort, fatigue and clumsiness during ADL task performance. Baseline mean AMPS ADL process ability measure was also below competence cut-off indicating inefficiency (e.g., disorganization), safety risk, and potential need for assistance in everyday life. Information on years living with a chronic condition was unavailable due to lack of access to medical records.

**Table 1:** Participant demographic data

Variable	Clients			OTs/OTSs	
	Total (*n* = 30)	Completers (*n* = 20)	Dropouts (*n* = 10)	OTs (*n* = 2)	OTSs (*n* = 5)
Gender: Female, *n* (%)	21 (70)	14 (70)	7 (70)	2 (100)	5 (100)
Age: Median^a^ (range)	72 (55–85)	73 (55–85)	70 (55–85)	52 (43–61)	23 (22–24)
Diagnosis ^b^: *n* (%)					
Neurologic	8 (27)	5 (25)	3 (30)		
Medical	12 (40)	8 (40)	4 (40)		
Musculoskeletal	10 (33)	7 (35)	3 (30)		
Civic status: *n* (%)					
Living alone	17 (57)	12 (60)	5 (50)		
Living with a partner	13 (43)	8 (40)	5 (50)		
Job situation: *n* (%)					
Working	1^c^ (3)	0	1^c^ (10)		
Sick leave	2 (7)	2 (10)	0		
Senior citizen	27 (90)	18 (90)	9 (90)		
Assistance in the home^d^: *n* (%)	26 (87)	19 (95)	7 (70)		
Spouse	8 (31)	5 (26)	3 (30)		
Relatives	16 (62)	11 (58)	5 (50)		
Friends	10 (38)	9 (47)	1 (10)		
Home care	11 (42)	8 (42)	3 (30)		
Hired house keeper/gardener	19 (73)	13 (68)	6 (60)		
Self-reported general health					
Median^a^ (range)	4 (3–5)	4 (3–5)	4 (3–5)		
Years as OT: Mean (range)				18 (16–20)	
Years working with clients with chronic conditions: Mean (Range)				18 (16–20)	
AMPS ADL Motor ability^e^: mean (SD)	0.92 (0.56)	0.93 (0.51)	0.91 (0.70)		
AMPS ADL Process ability^e^: mean (SD)	0.74 (0.37)	0.73 (0.38)	0.78 (0.38)		
ADL-I^f^: mean (SD)	2.69 (1.45)	2.62 (1.63)	2.83 (1.07)		

*AMPS* Assessment of Motor and Process Skills, *OTs* occupational therapists, *OTSs* occupational therapy students

^a^Based on median and range due to lack of normal distribution in data/due to ordinal data.

^b^Several of the clients had more than one diagnosis. The diagnoses listed above are the ones that the clients perceived to affect their everyday life the most

^c^One client was working 9 hours a week

^d^Clients could mark all relevant categories

^e^AMPS at baseline

^f^ADL-I at baseline

## Evaluation of feasibility

Results will be presented using the outline in Additional file 1 [17, 20] addressing selected aspects of feasibility: *intervention development*; *intervention components*; *mechanisms of action*; *perceived value*, *benefits*, *harms*, *and unintended consequences*; *feasibility and acceptability in practice*; and *fidelity*, *reach*, *and dose*.

### Intervention development and components

#### Objective: determine adjustments made to make the intervention program more acceptable and/or relevant

The OTs made some adjustments to enhance the acceptability and relevance of the intervention. Thus, the initial AMPS evaluation was not implemented with two (6.6%) clients; one client was too frail to participate and short after hospitalized and another client reported no ADL problems during the ADL-I interview and consequently left the program. At the final session, one (5%) client did not wish to participate in the AMPS re-evaluation. Similarly, the OTs were not able to set GAS goals with six (24%) clients; four clients did not express any goals and therefore left the program after session 2 and two clients did not want to/could not see the point in setting goals but continued in the program. Further, the OTs adjusted the number of sessions; only eight (40%) clients completing the program received the minimum dose (sessions 1, 2, and final, and a minimum of two intervention sessions). Reported reasons for this were that the clients did not have more goals to address.

#### Objective: identify specific components implemented including required time, equipment and materials

**Fig. 3:**
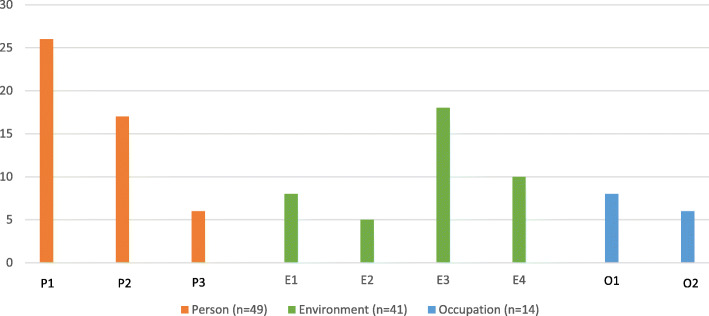
Frequency of implemented components throughout sessions 3–6 in the ABLE program. Abbreviations: P: Person; E: Environment; O: Occupation. P1: Changing habits related to task performance. P2: Changing attitude. P3: Plan, prioritize and reject. E1: Changing the physical environment. E2: Changing the social environment. E3: Use of tools, technology and/or helping aids. E4: Referring to other relevant services and opportunities. O1: Dividing the task into minor steps/distributing the task performance over longer time. O2: Simplifying the process/simplifying the task

**Fig. 4:**
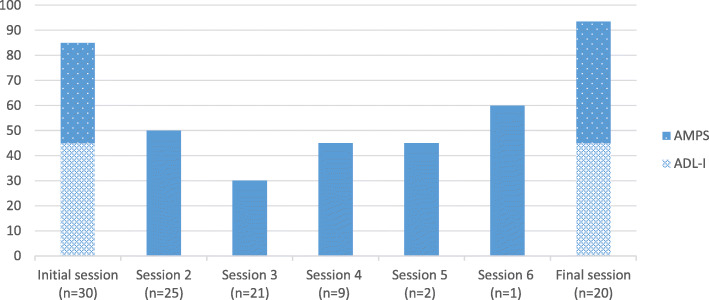
Median number of minutes used at each session. As no clients received session 7, the figure includes data from session 1–6 and the final session. *AMPS* Assessment of Motor and Process Skills, *ADL*-*I* ADL interview

All nine intervention components (Fig. 1) were applied during the intervention period. “Changing habits” (P1) related to task performance was the most frequently implemented component (*n* = 26) followed by “Using tools/technology/helping aids” (E3) (*n* = 18) and “Changing attitude” (P2) (*n* = 17) (Fig. 3). The median number of minutes spent at each session varied from 30 to 94 min across sessions with a tendency to spend more time on the first (median = 85 min) and final sessions (median = 94 min) involving evaluations (Fig. 4). In general, the OTs reported having access to needed equipment and materials during delivery. However, not having access to relevant helping aids to try out and practice with was reported as a problem 10 times during the intervention sessions.

### Mechanisms of action

#### Objective: Determine the extent to which intervention components contribute to goal achievement

Across the sessions, the clients reported that intervention components in a fair to high degree contributed to progress toward goal attainment. The OTs generally reported similar or slightly lower ratings (Table 2).

**Table 2:** Feasibility aspects based on registrations from occupational therapists, occupational therapy students, and clients

	Session 1	Session 2	Session 3	Session 4	Session 5	Session 6	Final session
OT/OTS: Number of registrations: *n* (%)	30 (100)	25 (100)	21 (100)	8 (89)	2 (100)	1 (100)	20 (100)
Intervention component support progress toward goals^a^	3 (1–5)	3 (1–5)	3 (1–5)	3 (2–5)	3.5 (2–5)	2 (–)	–
Client involvement^a^	4 (1–5)	3 (1–5)	3 (2–5)	3 (3–5)	3 (2–4)	2 (–)	4 (2–5)
Meaningfulness to client^a^	3 (1–5)	3 (1–5)	3 (1–5)	3 (3–5)	3 (2–4)	3 (–)	3 (1–5)
Meaningfulness to OT^a^	4 (2–5)	3 (1–5)	3 (1–5)	4 (3–4)	3.5 (3–4)	3 (–)	4 (3–5)
Confidence in delivery^a^	4 (3–5)	4 (3–5)	4 (2–4)	4 (2–4)	3.5 (3–4)	3 (–)	4 (4–5)
Clients: Number of registrations: *n* (%)	25 (83)	25 (100)	19 (90)	9 (100)	2 (100)	1 (100)	20 (100)
Intervention component support progress toward goals^a^	3 (1–5)	4 (2–5)	4 (1–5)	4 (1–5)	3,5 (3–4)	4 (–)	3,5 (1–5)
Being informed^a^	4 (2–5)	4 (3–5)	4 (1–5)	4 (1–5)	2,5 (1–4)	4 (–)	4 (1–5)
Being involved^a^	4 (3–5)	5 (3–5)	4 (1–5)	4 (1–5)	4 (3–5)	4 (–)	4 (1–5)
Meaningfulness^a^	4 (3–5)	4 (1–5)	4 (1–5)	4 (1–5)	4 (3–5)	3 (–)	4 (1–5)
Overall satisfaction^a^	4 (2–5)	4 (2–5)	4 (1–5)	4 (1–5)	4 (3–5)	3 (–)	4 (1–5)

As no clients received session 7, the table includes data from session 1–6 and the final session

All aspects are rated based on a VAS scale from 1 to 5; 1 = very low degree, 2 = low degree, 3 = fair degree, 4 = high degree, and 5 = very high degree

^a^Based on median and range as the VAS ratings are ordinal data.

**Fig. 5:**
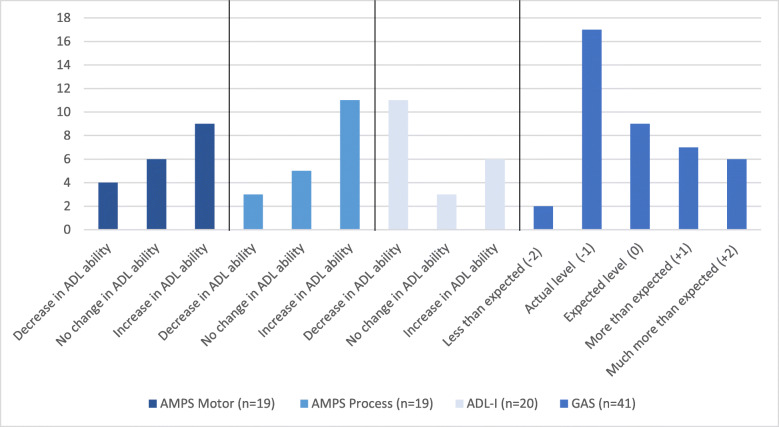
Responders and non-responders based on ADL-I, AMPS and GAS. *AMPS* Assessment of Motor and Process Skills, *ADL*-*I* ADL interview, *GAS* Goal Attainment Scaling

Eighteen clients, who set goals using GAS and completed ABLE 1.0, defined a total of 42 goals related to ADL task performance (median 2, range 1–4). Of these, 22 (52%) goals were reached as “expected,” “more than expected,” or “much more than expected,” whereas 17 (40%) goals were not reached. Re-evaluation data was missing for one goal (2%) (Fig. 5). Below an example of a client goal related to bathing is presented:

“Much more than expected” (level + 2): *Can take a bath several times during the week*—*no rest needed afterwards*

“More than expected” (level + 1): *Can take a bath two times during the week*—*no rest needed afterwards*

“Expected” (level 0): *Can take a bath two times during the week*—*need of a five min. rest afterwards*

“Unchanged/actual level” (level − 1): *Can take a bath two times during the week*—*need of a fifteen min. rest afterwards*

“Less than expected” (level − 2): *Can take a bath one time during the week*—*need of a fifteen min. rest afterwards*

#### Objective: determine the proportion of participants obtaining clinically relevant improvements in ADL ability

Sixteen (80%) of the clients completing ABLE 1.0 obtained a clinically relevant improvement in ADL ability (i.e., were responders) either based on self-report or observation. Of these, three (18.7%) achieved clinically relevant improvements based on both self-report and observation. More specifically, nine (45%) obtained clinically relevant improvements in ADL motor ability and eleven (55%) in ADL process ability. Further, six (30%) clients achieved a clinically relevant improvement based on ADL-I measures (Fig. 5). All responders were senior citizens and on average nine years older than the non-responders. Further, the responders’ mean AMPS ADL motor ability measure at baseline was 0.41 logits lower compared to non-responders, representing a clinically relevant difference (> 0.30 logits) in ADL ability [32] (Table 3).

**Table 3:** Demographic data on responders and non-responders

Variable	Responders based on AMPS/ADL-I (*n* = 16)	Non–responders (*n* = 4)
*Gender:* Female, *n* (%)	11 (69)	3 (75)
*Age*: Median^a^ (Range)	74 (55–85)	65 (60–80)
Diagnosis^b^: *n* (%)		
Neurologic	4 (25)	1 (25)
Medical	7 (44)	1 (25)
Musculoskeletal	5 (31)	2 (50)
Civil status: *n* (%)		
Living alone	9 (56)	3 (75)
Living with a partner	7 (44)	1 (25)
Job situation: *n* (%)		
Sick leave		2 (50)
Senior citizen	16 (100)	2 (50)
Assistance in the home^c^: *n* (%)	15 (94)	4 (100)
Spouse	4 (27)	1 (25)
Relatives	8 (53)	3 (75)
Friends	8 (53)	1 (25)
Home care	7 (47)	1 (25)
Hired housekeeper/gardener	11 (73)	2 (50)
Self-reported general health^d^		
Median^a^ (range)	4 (3-5)	4 (3-5)
ADL Motor ability^e^: mean (SD)	0.84 (0.51)	1.25 (0.39)
ADL Process ability^e^: mean (SD)	0.68 (0.30)	0.93 (0.62)
Received number of sessions		
Median^a^ (range)	4 (4–7)	4 (4–5)

^a^Based on median and range due to lack of normal distribution in data or due to ordinal data

^b^Several of the clients had more than one diagnosis. The diagnoses listed above are the ones that the participants perceived to affect their everyday life the most.

^c^Clients could mark all relevant categories

^d^SF1 at baseline

^e^AMPS at baseline

### Perceived value, benefits, harms, and unintended consequences of the intervention

#### Objectives: determine the most beneficial intervention components and the extent to which the components are perceived meaningful

The clients overall reported a high to very high degree of being informed and involved (Table 2), whereas the OTs generally reported slightly lower levels of client involvement. In addition, the clients stated a fair to high degree of meaningfulness and satisfaction with the content of single sessions (Table 2). Again, when the OTs were asked if the content of session was meaningful to the client, their median ratings were slightly lower than client ratings. Still, the OTs reported a fair to high degree of perceived professional meaningfulness of content across sessions.

#### Objective: identify unintended positive/negative side effects

The clients reported no positive/negative side effects. Instead, seven (35%) clients expressed that participation in ABLE 1.0 overall was positive. The OTs reported some positive side effects, e.g., *“Since last session, the client has initiated making appointments (doctor/masseuse)”* and *“the client is happy—she has taught two friends how to change sheets using the method I have taught her.”*

### Feasibility and acceptability of intervention in practice

#### Objective: determine the retention rate and if the program seems to be feasible across sub-groups

Nine (30%) clients dropped out after sessions 1 or 2 (i.e., before receiving any interventions) and one client after session 4 (3.3%) mainly due to lack of perceived need and/or motivation for intervention. The remaining clients (*n* = 20) received one or more intervention session and completed re-evaluation (Fig. 2). The demographic and general health data on clients completing the program (completers) and clients who dropped out (dropouts), respectively, was explored in post-hoc descriptive analyses (Table 1). When comparing mean AMPS ADL ability measures across the two groups, no differences in observed ADL ability were seen. Also, 27 (96%) clients who participated in the AMPS at the initial session had AMPS ADL motor ability measures below the competence cut off at 2.0 logits, suggesting a potential need for intervention.

#### Objective: describe challenges, satisfaction, and confidence in relation to delivering the intervention

Overall, the OTs perceived a fair to a high degree of confidence in delivering the intervention (Table 2). At the same time, they reported that their limited experience with the program was affecting the delivery. For example, one of the OTs reported:* “I need more experience to let go of old ways of doing.”* With regards to challenges, several notes were made, e.g., “*the initial session was time consuming and therefore demanding for the client”*; *“the client had high expectations related to exercising and provision of helping aids”*; *“the client had a lot of disturbing pets”*; *“the client’s home was very crowded”*; *“the time of year limited outdoor activities”* and *“the client had economic limitations and could not afford buying minor helping aids”*; *“the client had just moved in and had a lot of other things on the mind.”*

#### Objective: identify institutional/organizational facilitators and barriers during delivery

Few notes (*n* = 5) were made related to institutional/organizational barriers during delivery. The notes concerned the ineffective procedures related to obtaining access to helping aids when needed, e.g., “*having to beg and argue—in writing—and having to follow up on my request merely to get access to a reacher*.” No notes concerned institutional/organizational facilitators.

### Fidelity, reach, and dose of intervention

#### Objective: determine adherence to intervention procedures and manual

Overall, OTs’ adherence to the manual was high. Aside from previously mentioned deviations, OTs only reported minor adjustments, all within the frame of the guidelines described in the manual. One of the OTs noted that “the manual definitely supports the process,” suggesting that the manual was used to guide the intervention.

#### Objective: determine the number of sessions for each participant and duration of each session

During the study period, 108 sessions were delivered; five (5%) by telephone. The number of sessions per client completing the program was median = 4 (range 4–7). For number of minutes spend at each session, see Fig. 4.

#### Objective: determine if each participant had a sufficient dose

According to the OTs’ registrations, 16 (80%) clients completing the program had a sufficient dose, whereas a few more intervention sessions would have been beneficial in four cases. One (5%) client noted that duration of the entire program was too long. Otherwise, the clients were satisfied with duration of the program.

## Discussion

In this study, we evaluated the feasibility of the ABLE 1.0 program. By addressing aspects of *intervention development*, *intervention components*, *mechanisms of action*, *perceived value*, *benefits*, *harms or unintended consequences*, *feasibility and acceptability in practice and fidelity*, and *reach and dose of intervention* results revealed that content and delivery of the program was overall feasible. However, adjustments are needed in relation to the recruitment procedure, including recruiting clients with a perceived need for addressing ADL task performance problems. Further, minor adjustments to the program manual are needed. Below findings will be discussed in more detail and specific adjustments will be suggested.

### Recruitment and retention

Existing research indicate that persons living with a chronic condition experience decreased ADL ability [2–8]. Consequently, the ABLE 1.0 program was developed. Still, when recruiting clients for this feasibility study, 35% of the persons contacted declined participation, reporting lack of perceived need or motivation. One reason could be that persons, who had already received a standard diagnosis-specific, group-based rehabilitation programs, were less motivated for additional intervention, suggesting that recruitment for the ABLE program should be earlier in the clients’ rehabilitation process, potentially before or simultaneously with other programs. Another reason could be discrepancy between what was presented about the intervention by the recruiter and the person’s perceived needs.

Based on the recruitment strategy, clients representing variation in diagnoses, gender, and age were included. Their observation-based ADL ability measures at baseline suggested need for intervention. Especially AMPS ADL motor ability measures below the competence cut off indicated increased physical effort, clumsiness, and fatigue during ADL task performance. Despite of this, more than a quarter of the clients dropped out before session 3 reporting no need or lack of motivation for intervention. When comparing mean AMPS ADL ability measures between those who completed the program and those who dropped out, no differences in observed ADL ability were seen. This suggests other reasons for drop out, e.g., discrepancy between what the clients expected and what they received.

So, despite thorough introduction of both recruitment personnel and OTs, a stronger link between what is presented to potential participants during the recruitment process about the actual content of the ABLE program and how it is delivered, may be needed.

### Intervention: adherence and content

Most clients completing the program went through interview- and observation-based ADL evaluations in the first session, forming the basis for goal setting and intervention sessions, before re-evaluation. In line with previous findings [46], sessions involving evaluations were the most time consuming, whereas the intervention sessions overall were shorter. Still, initial evaluation sessions involving both a formal ADL interview (ADL-I) and a standardized observation (AMPS) conducted within less than 1.5 h by the OTs, and a little more than 1.5 h by OTSs, seems reasonable. The findings may also indicate that a thorough initial evaluation provides a solid foundation for planning and implementing interventions, potentially reducing time used on intervention sessions. The relevance of the initial session was stressed by the fact that both clients and OTs found these evaluations highly meaningful and supportive of client involvement in the process. Also, the OTs felt highly confident in delivering this session.

The OTs made minor adjustments to the content of the program based on the clients’ needs, hence reflecting the client-centered, individualized approach in the program [24]. For example, in a couple of cases, the clients completing the program could see no point in participating in goal setting. Goal setting is known to be challenging, both to clients and professionals [47–51]. Still, the clients overall found the goal setting session highly meaningful, supporting this session to stay mandatory.

Overall, both clients and OTs reported that intervention components applied in a fair to high degree supported progress toward goal attainment. Each of the nine intervention components were applied at least five times during the intervention period, supporting their relevance. “Changing habits related to task performance” (P1) was the most frequently implemented intervention component. As most adaptational strategies involve some kind of habit change, there might have been overlaps in the intervention components making it hard for the OTs to distinguish between them and to mark the one/the ones used. Further, there is no clear definition of how much the OT has to do, to justify that a component has been applied. Thus, there may be a need to clarify differences between components and define what needs to be done for a component to be considered applied. Consequently, a revised version of the intervention manual will be developed.

### Dose and potential outcomes

Intervention dose is an important factor potentially influencing the outcome [52, 53]. In a recent study among persons with advanced cancer [52], a low intervention dose (one home visit and one follow up telephone contact) potentially resulted in little /no effect of the occupational therapy intervention. Similar findings were revealed in a study among persons with Parkinson’s disease receiving a median dose of four sessions over 8 weeks [53]. In the present study, only 40% of the clients, who remained in the program, received the defined minimum dose (five sessions); after initial evaluation and goalsetting, most of them only needed a single intervention session to address their goals. Despite that most of the clients only received one intervention session, the majority (80%) of the clients completing the program obtained a clinically relevant improvement in ADL ability. Thus, reducing the minimum dose to four sessions (sessions 1–3 and final session) might be relevant. Also, since almost half of the identified goals were not attained, it is worth considering keeping a maximum dose of up to eight sessions.

Overall, the clients obtaining a clinically relevant improvement in ADL ability (responders) were 10 years older than the non-responders and had a clinically relevant lower observation-based ADL ability at baseline, suggesting more room for improvement. As ADL ability decreases with age [32], one possible reason for the lower ADL ability among the responders simple could be that they were older. Still, based on the present data, it cannot be determined if age or level of ADL ability at baseline are markers of who will benefit of the ABLE program. Moreover, while the non-responders had a clinically relevant higher observation-based ADL ability at baseline, their mean ADL motor and ADL process ability measures still were below competence cut-offs demonstrating a likely need for intervention.

Since a limited relationship between self-reported and observed ADL ability is well established [2, 3], outcome measures based on both self-report (ADL-I) and observation (AMPS) were applied in the present study. The findings confirmed this pattern; very few clients gained an increase in both self-reported and observed ADL ability. Consequently, the initial and final evaluation sessions in ABLE will continue to include both interview- and observation-based evaluation of ADL ability.

### Context of delivery

Interestingly, these quite experienced OTs reported a high degree of confidence in delivering the program across sessions, but at the same time commented that they needed more experience with the program. This contrast will be further explored based on the data gathered in the qualitative interviews.

The second most employed intervention component was “Using tools/technology/helping aids.” Still, the OTs reported limited access to or ineffective procedures for obtaining helping aids to use for practicing with the clients. As implementation of adaptive equipment or assistive technology is a central aspect of the compensatory model in the OTIPM [24], it is important to ensure access to such equipment, in future studies of the ABLE program.

### Strengths and weaknesses of the study

A strength was that the intervention was developed based on a structured process integrating evidence from various sources and framed by occupational therapy theory. Moreover, the study was conducted based on and in accordance with a published protocol [17] ensuring transparency of the research. There were no differences between the protocol and the study conducted. Both quantitative and qualitative feasibility data representing the perspectives of the OTs/OTSs, and clients was collected. As a result, broad perspectives related to the content and delivery were evaluated.

The present study also had some limitations. First, the sample size was small, and limited by a rather large drop-out due to hospitalization, lack of motivation or lack of perceived need. In addition, this study was conducted in just one setting with few OTs involved in delivering the program increasing the risk of not uncovering all challenges in the program.

## Conclusion

Overall, ABLE 1.0 was feasible in terms of content and delivery, when delivered in Danish municipality. More specifically, the program was feasible with regards to *intervention development*, *intervention components*, *mechanisms of action*, *perceived value*, *benefits*, *harms or unintended consequences*, *feasibility and acceptability in practice and fidelity*, and *reach and dose of intervention.* However, the study revealed a need to make minor changes in the intervention manual and adjust the recruitment procedure before a future RCT study. The focus of this feasibility study was on content and delivery of ABLE 1.0. To evaluate other aspects of feasibility in terms of design, conduct and processes of an outcome trial, including recruitment, randomization, adherence, and how to measure potential outcomes, it would be relevant to proceed by planning and conducting a pilot RCT study [54].

## Supplementary Information

**Additional file 1.**

**Additional file 2.**

**Additional file 3.**

## Data Availability

The datasets used and/or analyzed during the current study are available from the corresponding author on reasonable request.

## Abbreviations

ABLE: A Better Everyday LifE
ADL: Activities of daily living
ADL-I: Activities of daily living interview
AMPS: Assessment of Motor and Process Skills
GAS: Goal Attainment Scaling
IADL: Instrumental activities of daily living
OTs: Occupational therapists
OTSs: Occupational therapy students
OTIPM: Occupational Therapy Intervention Process Model
PEO model: Person-Environment-Occupation model
PADL: Personal activities of daily living
RCT: Randomized controlled trial
SD: Standard deviation
VAS: Visual analog scale
