# Supplementary material for: Feasibility of ABLE 1.0—a program aiming at enhancing the ability to perform activities of daily living in persons with chronic conditions
Source: Pilot Feasibility Stud. 2021 Feb 18;7:52. doi: 10.1186/s40814-021-00790-7 (PMC7891027; doi:10.1186/s40814-021-00790-7)
Supplement: Supplementary file 1 — Additional file 1. [file 40814_2021_790_MOESM1_ESM.docx]

Additional file 1: Overview of the specific objectives and related data collection methods based on the framework suggested by O’Cathain

| Sub-categories | Intervention development | Intervention components | Mechanisms of action | Perceived value, benefits, harms or unintended consequences of the intervention | Acceptability of intervention in principle | Feasibility and acceptability of intervention in practice | Fidelity, reach and dose of intervention |
| --- | --- | --- | --- | --- | --- | --- | --- |
| Specific objective | Determine adjustments made to make the intervention program more acceptable and/or relevant in the specific context. | Identify specific components implemented, including required time, equipment and material.  Determine adjustments made to make the specific component more acceptable and/or relevant in the specific context. | Determine the extent to which intervention components contribute to goal achievement.  Determine the proportion of participants obtaining clinically relevant improvements in self-reported and/or observed ADL ability. | Determine the most beneficial intervention components.  Identify unintended positive/negative side effects.  Determine the extent to which the components are perceived meaningful. | Evaluate the overall perception of the content and delivery of the program.  Determine to which extent the program has potential to be implemented in usual practice. | Determine the retention rate and if the program seems to be feasible across e.g. gender and diagnostic groups.  Describe challenges, satisfaction and confidence in relation to delivering the intervention.  Identify institutional/ organizational facilitators and barriers during delivery. | Determine adherence to intervention procedures and manual.  Determine the number of sessions for each participant and duration of each session.  Determine if each participant had a sufficient dose. |
| Data collection method | Registration forms will be filled out by the occupational therapists after each session in the program. | Registration forms will be filled out by the occupational therapists after each session in the program. | Registration forms will be filled out by occupational therapists and participants after each session in the program.  Goal setting  Pre and post assessment of ADL ability. | Qualitative interviews with occupational therapists and participants after completing the data collection for both participant groups.  Registration forms will be filled out by occupational therapists and participants after each session in the program. | Qualitative interviews with occupational therapists and participants after completing the data collection for both participant groups. | Questionnaire on the participants’ demographic data.  Registration forms will be filled out by the occupational therapists after each session in the program. | Registration forms will be filled out by the occupational therapists after each session in the program. |
| Data | Registrations of deviations from the intervention manual. | Registrations of applied components, time use, and need equipment.  Registrations of deviations from the intervention manual. | GAS  ADL-I  AMPS | Transcribed interview data  Registrations on unintended positive/negative side effects  and perceived degree of meaningfulness. | Transcribed interview data | Demographic data  Registrations on retention, challenges, satisfaction, confidence and institutional/ organizational facilitators and barriers. | Registrations of number of sessions for each participant, time use and deviations from the intervention manual. |

ADL-I, ADL Interview, version 2; AMPS, Assessment of Motor and Process Skills; GAS, Goal Attainment Scaling.
