# Supplementary material for: Feasibility of ABLE 1.0—a program aiming at enhancing the ability to perform activities of daily living in persons with chronic conditions
Source: Pilot Feasibility Stud. 2021 Feb 18;7:52. doi: 10.1186/s40814-021-00790-7 (PMC7891027; doi:10.1186/s40814-021-00790-7)
Supplement: Supplementary file 2 — Additional file 2. [file 40814_2021_790_MOESM2_ESM.docx]

**Additional file 2. Example of a registration form for occupational therapists.**

**Registration form. A Better everyday LifE (ABLE) - Session 3-7: Intervention**

Occupational therapist:

Client ID code:

Date: Session no.:

Mark the component(s) implemented in this session:

| **Intervention components** | **Notes about the specific session** | **Duration** | **Did you follow the manual?** | **If not – what did you do differently?** |
| --- | --- | --- | --- | --- |
| □ Face to face contact  □ Telephone contact |  |  |  |  |
| **Changes related to person (P)**  □ P1. Changing habits related to ADL task performance  □ P2. Changing attitude  □ P3. Planning, prioritizing and rejecting  **Changes related to environment (E)**  **□** E1. Changing physical environment  **□** E2. Changing social environment  **□** E3. Using tools, technology and/or helping aids  □ E4. Referring to other relevant services and opportunities  **Changes related to occupation (O)**  □ O1. Dividing the ADL task performance into minor parts/ Distributing the ADL task performance over a longer period of time  □ O2. Simplifying the process/simplifying the ADL task |  |  | □ Yes  □ No  □ Yes  □ No  □ Yes  □ No |  |

Please, rate below using a scale from 1-5.

| Question: | 1= to a very low degree | 2= to a low degree | 3= to a fair degree | 4= to a high degree | 5= to a very high degree |
| --- | --- | --- | --- | --- | --- |
| To which extent did you feel confident during this session? |  |  |  |  |  |
| To which extent did you involve the client? |  |  |  |  |  |
| To which extent did this session support progress towards the client’s goal(s)? |  |  |  |  |  |
| To which extent do you think that this session was meaningful to the client? |  |  |  |  |  |
| To which extent do you think that this session was meaningful to you? |  |  |  |  |  |

Did you experience any positive/negative side effects during based on this session? If yes, which?

Did you experience practical and/or organizations conditions facilitating/hindering the delivery of this session? If yes, which?

Did you have access to the needed tools or materials during this session? If no, what did you need?

Other comments:
