# Supplementary material for: Feasibility of ABLE 1.0—a program aiming at enhancing the ability to perform activities of daily living in persons with chronic conditions
Source: Pilot Feasibility Stud. 2021 Feb 18;7:52. doi: 10.1186/s40814-021-00790-7 (PMC7891027; doi:10.1186/s40814-021-00790-7)
Supplement: Supplementary file 3 — Additional file 3. [file 40814_2021_790_MOESM3_ESM.docx]

**Additional file 3. Example of a registration form for clients.**

**Registration form. A Better everyday LifE (ABLE) - Session 3-7: Intervention**

Client:

Date:

Please, rate below using a scale from 1-5.

| Question: | 1= to a very low degree | 2= to a low degree | 3= to a fair degree | 4= to a high degree | 5= to a very high degree |
| --- | --- | --- | --- | --- | --- |
| To which extent did you feel informed during this session? |  |  |  |  |  |
| To which extent did you feel involved during this session? |  |  |  |  |  |
| To which extent did you feel that this session provided progress towards your goal(s)? |  |  |  |  |  |
| To which extent did you find this session meaningful? |  |  |  |  |  |
| To which extent did you find this session satisfying? |  |  |  |  |  |

| **Other comments related to this session:** |
| --- |
|  |
